# Left Atrial Appendage Closure Guided by Integrated Echocardiography and Fluoroscopy Imaging Reduces Radiation Exposure

**DOI:** 10.1371/journal.pone.0140386

**Published:** 2015-10-14

**Authors:** Christiane Jungen, Tobias Zeus, Jan Balzer, Christian Eickholt, Margot Petersen, Eva Kehmeier, Verena Veulemans, Malte Kelm, Stephan Willems, Christian Meyer

**Affiliations:** 1 Department of Cardiology—Electrophysiology, cNEP, cardiac Neuro- and Electrophysiology research group, University Heart Center, University Hospital Hamburg-Eppendorf, Hamburg, Germany, DZHK (German Center for Cardiovascular Research), partner site Hamburg/Kiel/Luebeck, Hamburg, Germany; 2 Department of Cardiology, Pulmonology and Vascular Medicine, Medical Faculty, University Hospital Duesseldorf, Duesseldorf, Germany; University of Messina, ITALY

## Abstract

**Aims:**

To investigate whether percutaneous left atrial appendage (LAA) closure guided by automated real-time integration of 2D-/3D-transesophageal echocardiography (TEE) and fluoroscopy imaging results in decreased radiation exposure.

**Methods and Results:**

In this open-label single-center study LAA closure (Amplatzer^TM^ Cardiac Plug) was performed in 34 consecutive patients (8 women; 73.1±8.5 years) with (n = 17, EN+) or without (n = 17, EN-) integrated echocardiography/fluoroscopy imaging guidance (EchoNavigator^®^ [EN]; Philips Healthcare). There were no significant differences in baseline characteristics between both groups. Successful LAA closure was documented in all patients. Radiation dose was reduced in the EN+ group about 52% (EN+: 48.5±30.7 vs. EN-: 93.9±64.4 Gy/cm^2^; p = 0.01). Corresponding to the radiation dose fluoroscopy time was reduced (EN+: 16.7±7 vs. EN-: 24.0±11.4 min; p = 0.035). These advantages were not at the cost of increased procedure time (89.6±28.8 vs. 90.1±30.2 min; p = 0.96) or periprocedural complications. Contrast media amount was comparable between both groups (172.3±92.7 vs. 197.5±127.8 ml; p = 0.53). During short-term follow-up of at least 3 months (mean: 8.1±5.9 months) no device-related events occurred.

**Conclusions:**

Automated real-time integration of echocardiography and fluoroscopy can be incorporated into procedural work-flow of percutaneous left atrial appendage closure without prolonging procedure time. This approach results in a relevant reduction of radiation exposure.

**Trial Registration:**

ClinicalTrials.gov NCT01262508

## Introduction

Percutaneous left atrial appendage (LAA) closure is currently under investigation as a promising catheter-based approach for stroke prevention in patients with atrial fibrillation (AF) [1,2,3]. This is important since the LAA is the source of thrombi in >90% of affected patients with nonvalvular AF [4] while oral anticoagulation still bears several limitations including bleeding risk [2]. Importantly, LAA closure still remains technically challenging [5]. Recently, a novel system enabling integrated echocardiography and fluoroscopy imaging (EchoNavigator^®^ [EN]), Philips Healthcare), which might at least partly overcome these limitations including radiation exposure, has been introduced [6,7]. The EN integrates in real-time information from 2D-/3D-transesophageal echocardiography (TEE) and fluoroscopy in the same anatomical alignment enabling improved visualization of catheters, guidewires and devices in relation to relevant anatomical structures [6]. The usefulness of this novel imaging approach during LAA closure procedures has not been systematically investigated so far. Herein, we investigated the utility of LAA closure guided by integrated echocardiography and fluoroscopy imaging. We hypothesized that this approach decreases radiation exposure.

## Methods

The protocol for this trial and supporting CONSORT checklist are available as supporting information (S1 CONSORT Checklist and S1 Protocol).

### Study design

**Fig 1:**
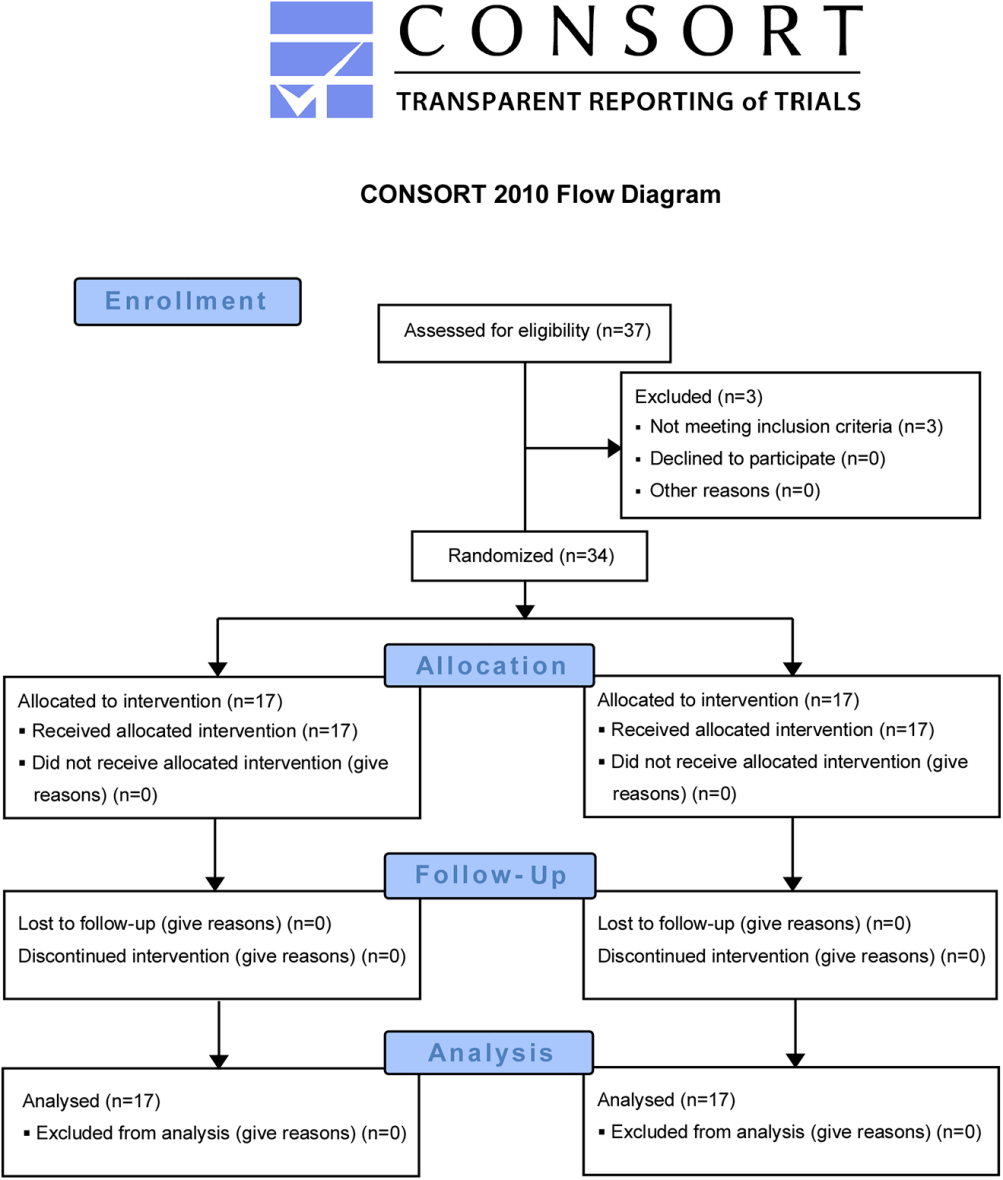
CONSORT flow chart.

In this open-label single-center study patients with nonvalvular AF, a CHA_2_DS_2_-VASc score of ≥1, a relative contraindication to oral anticoagulation, and a life expectancy of at least 2 years [5] were assigned by a computer software to LAA closure with (EN+) or without (EN-) the guidance of automated real-time integration of 2D-/3D- TEE and fluoroscopy imaging (Fig 1).

The *primary endpoint* of this study was the change of total radiation dose. The *secondary endpoints* were changes of fluoroscopy time, procedure time and contrast media amount. Successful LAA closure (residual flow <5mm) and acute (7-day) occurrence of death, ischemic stroke, systemic embolism and procedure or device related complications requiring major cardiovascular or endovascular intervention were determined [8,9].

The ethics review committee of the Heinrich-Heine-University Duesseldorf (Ethics review committee of the medical faculty, building 13.41, Moorenstrasse 5, 40225 Duesseldorf, Germany) approved this study and written informed consent was given by each patient. Patients undergoing LAA closure between February 2012 and March 2014 were included. Philips did not influence study design, data analysis, or manuscript preparation.

### LAA closure

Percutaneous catheter-based LAA closure in all patients was performed with the Amplatzer^TM^ Cardiac Plug (ACP) (AGA-St-Jude, Minneapolis, MN, USA) under conscious sedation by using boluses of midazolam and a continuous infusion of propofol (2%). A TEE probe was introduced to rule out intracardiac/LAA thrombus. An initial bolus of unfractionated heparin (80–100 IU/kg of body weight) was administered prior to a single transseptal puncture by using the modified Brockenbrough technique (LAMP^TM^, 45°, SWARTZ^TM^, St. Jude Medical^®^, St. Paul, USA; BRK^TM^, SJM) under TEE control. The heparin dose was adjusted during the procedure in all patients to achieve an activated clotting time >300 seconds. LAA orifice diameter and landing zone were measured by using 2D-TEE (mid-esophageal view at 0°, 45°, 90°, 135°) and angiography (right anterior oblique 30/25 caudal and right anterior oblique 30/15 cranial). A 13 French delivery sheath (45°x45° Amplatzer TorqVue^®^, SJM) was used for LAA angiography and ACP positioning. The device was chosen to be 10–20% larger than the maximum diameter of the LAA [2]. The ACP lobe was placed 1mm from the LAA orifice with the device’s long axis being positioned parallel to the LAA wall with the disc of the ACP aligned with the plane of the LAA orifice. After performing a tug test to ensure a secure fit, the device was released. A waiting period of at least 10 minutes was used to confirm a stable position of the LAA occluder. Clopidogrel and acetylsalicylic acid were prescribed for 3 months, followed by acetylsalicylic acid intake alone.

### Integrated echocardiography and fluoroscopy imaging: The EchoNavigator

**Fig 2:**
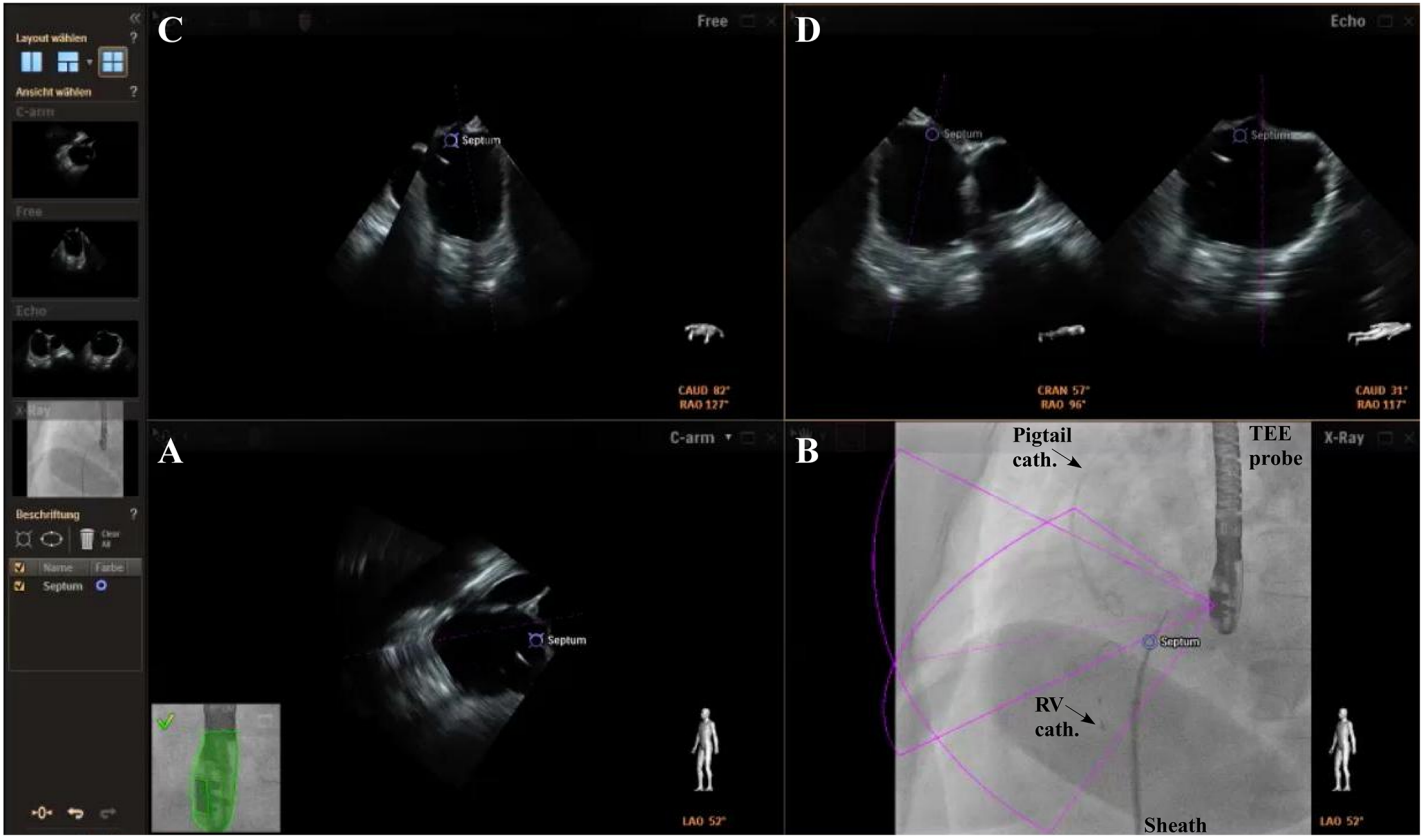
Overview of integrated echocardiography and fluoroscopy imaging. The image acquisition angles during transseptal puncture are depicted in the lower right corner of each image. A+B) Concordant views of TEE and fluoroscopy images. C) In the “Free view” echocardiographic images can be rotated and zoomed independently from the echocardiographer by using a tableside control. D) Conventional echocardiographic view using the x-plane mode for identification of the preferred transseptal puncture site (Septum, blue). Sheath with transseptal needle; Pigtail cath. = pigtail catheter; RV cath. = catheter in the right ventricle.

**Fig 3:**
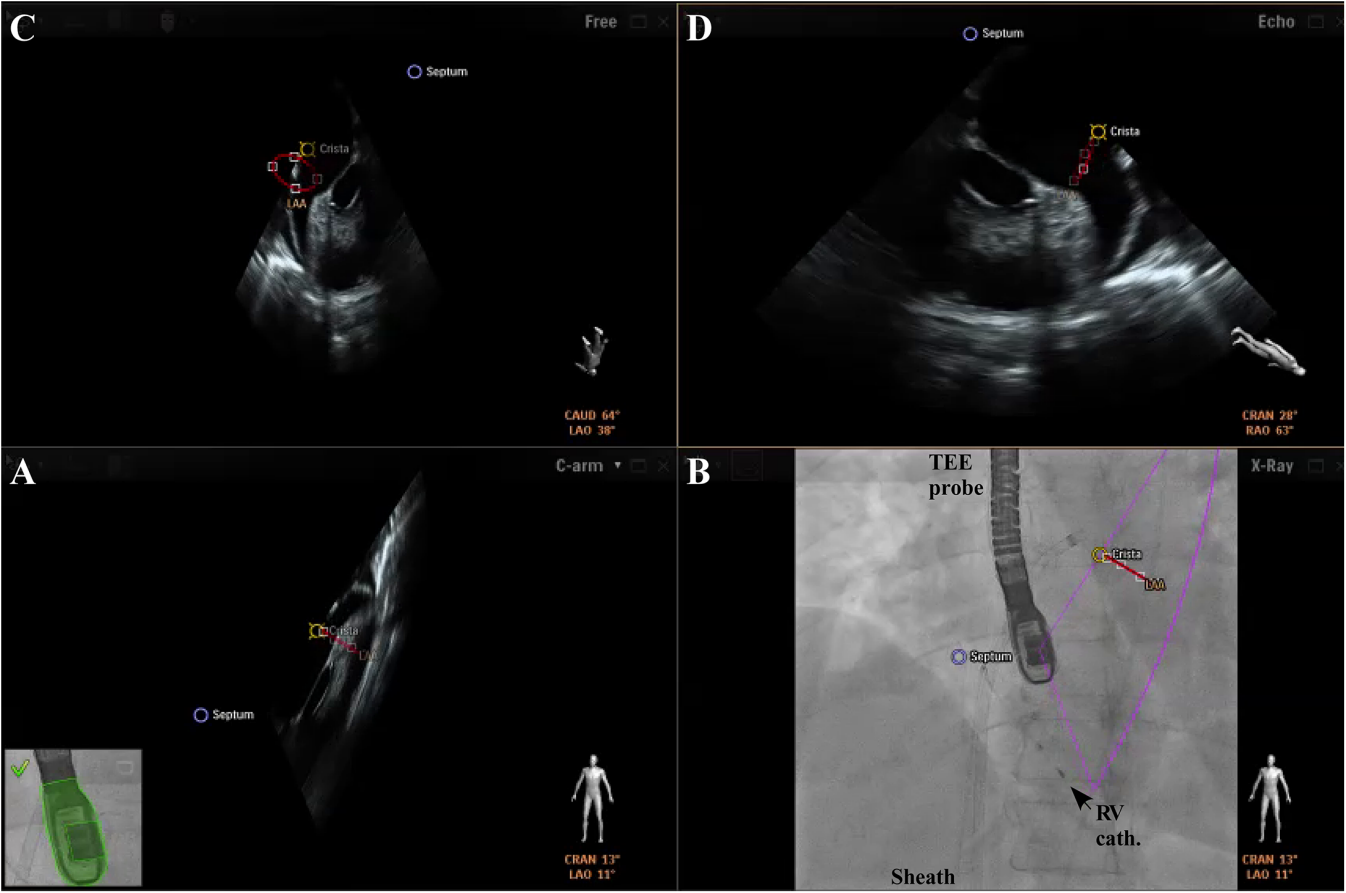
Visualization of the LAA and surrounding structures preceding occluder positioning. The LAA orifice (red), the crista (yellow), and the septum (blue) are marked by landmark setting. A) 2D-TEE image in the same anatomical alignment as the C-arm. B) Outlines of 2D-TEE (pink lines) are fused with the fluoroscopy image. C+D) 2D-TEE with anatomical landmarks depicted in different angles. Note the sheath in close proximity to the left superior pulmonary vein as supported by the matching landmarks in different views. RV cath. = catheter in the right ventricle.

**Fig 4:**
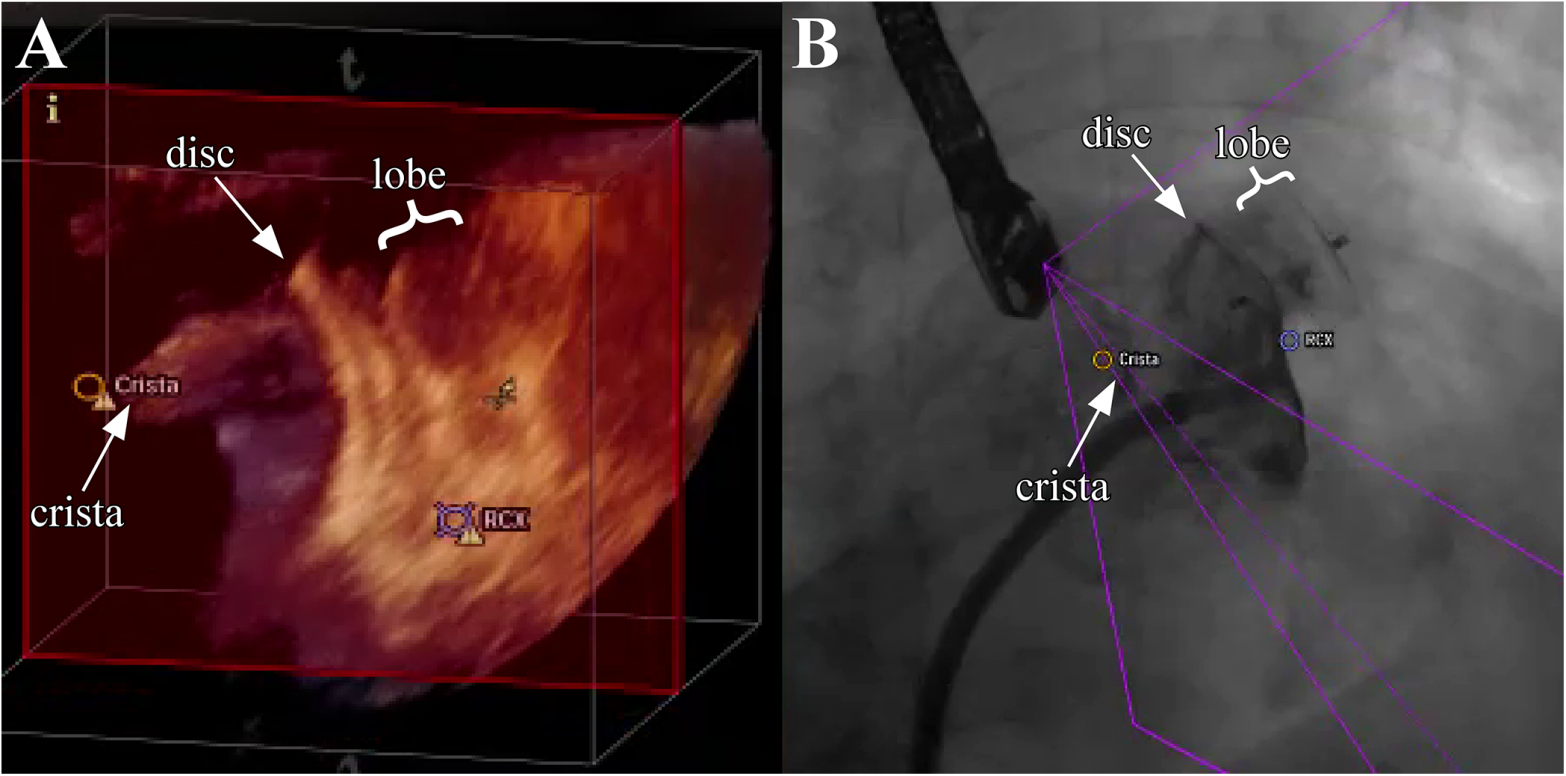
Evaluation of adequate device position and stability by using 3D-TEE and fluoroscopy. After LAA occluder release correct positioning is verified simultaneously by rotation and zoom of the 3D-TEE image and angiography. The LAA occluder is shown in the 3D-TEE “Free view” (A) by using the tableside control and the fluoroscopy (B) demonstrating the relationship to surrounding structures (LCX, crista). Note the relatively large crista which could not be fully covered by the disc of the LAA occluder, while contrast agent injection demonstrated good LAA sealing.

For automated real-time integration of echocardiography and fluoroscopy imaging a novel image integration system, the EN (Philips Healthcare, Best, The Netherlands), has been introduced. The system enables presentation of echocardiographic images in the same anatomical alignment as the C-arm of the fluoroscopy unit (“C-arm” view) so that both images of 2D-/3D-TEE and fluoroscopy (“X-Ray” view) are concordant in size and orientation in 3D space [6]. Changes in angulation, rotation, or position of the TEE probe are immediately registered and updated on fluoroscopy images [6]. Movement of the C-arm induces updates of the echocardiography images with the same angulation. In a third window, the interventional cardiologist can simultaneously rotate and zoom an echocardiography image (“Free” view) independently from the echocardiographer by using a tableside control. In a fourth window, conventional echocardiographic views (“Echo” view) are available. Each separate image display can further be modified by using features like contrast, panning or cropping. Depending on operator’s preferences two, three, or four windows are presented (Fig 2). Additionally, a “marker feature” (Fig 3) allows setting markers on typical anatomical regions in the echocardiography image. These markers are simultaneously displayed in real-time on the fluoroscopy image. This feature is helpful to recognize anatomical landmarks including the interatrial septum, the LAA orifice, the so called ridge (crista) between LAA and left pulmonary veins, and/or the left circumflex artery (LCX). These markers have been introduced to support orientation in general and to facilitate catheter and device guidance during live fluoroscopy. Usually, labelling of soft tissue targets takes place in the echocardiographic image, which is then displayed on the other image modality, e.g. in fluoroscopy. When the gantry is moved all markers are automatically updated on the fluoroscopy image without obvious lag time or need for re-registration. During LAA closure procedures this approach might support (1.) transseptal puncture, (2.) anatomical orientation and device positioning, and (3.) verification of adequate sealing and device stability. Therefore, depending on the interventionalist’s choice, the transseptal puncture was facilitated after setting a marker at the optimal puncture site by using the x-plane feature allowing simultaneous demonstration of two orthogonally orientated echocardiographic image planes (Fig 2). Afterwards, the EN was used for anatomical orientation in the left atrium and for positioning of the occluder within the LAA (Fig 3) following LAA measurements. The LAA orifice and additional anatomical landmarks were marked and optimal device positioning was evaluated in different angles simultaneously by using the C-Arm-, X-Ray-, Free-, and/or Echo view. After device release correct positioning was verified in 2D-/3D-TEE combined with color doppler and fluoroscopy/angiography (Fig 4) [10].

### Echocardiographic analysis

Echocardiographic measurements were performed in accordance with the guidelines of the American Society of Echocardiography [11] as described in detail before [12]. Obtained 2D-/3D-TEE images were stored in cine-loop format and analyzed in Xcelera R3.2L1 (Version 3 2011, Philips Medical Systems, Best, Netherlands). 3D-TEE images were recorded as 3D zoom dataset and analyzed with the multiplanar reconstruction mode in QLAB-3DQ (Version 8, Philips Medical Systems, Best, Netherlands) [13,14,15,16]. Left atrial diameter and area as well as calculation of the left ventricular ejection fraction were performed by using the Simpson biplane method [17,18]. We also compared several LAA measurements in different imaging methods (S1 Table and S1 Fig). In 2D-TEE the maximum and minimum diameter of the LAA orifice were obtained in orthogonal planes and measured as previously recommended [14]. The LAA orifice area was then calculated by using the equation [19]: LAA orifice area = π x (D_max_/2) x (D_min_/2). For comparison with 3D-TEE derived data, the larger of the two calculated orifice areas was used [13].

### Statistical analyses

Continuous variables are presented as mean values with standard deviation (SD) and categorical variables as numbers. For comparisons between groups with continuous variables an unpaired t-test was used. To compare differences across subgroups with not normally distributed data we used the Mann-Whitney test. To compare multiple subgroups of normally distributed data a one-way ANOVA was used. All analyses were performed using Prism 5 (GraphPad Prism 5.0, GraphPad Software Inc., San Diego, USA). As reported by prior studies showing a significant reduction of fluoroscopy exposure, we assumed a reduction by 10% compared with conventional procedures. The prospectively calculated sample size using 2-sided *t*-test analysis aiming for a power of 95% and an alpha of 0.05 was 14 patients per group. With a drop-out rate of 15% we assumed a group size of 17 patients.

## Results

### Baseline characteristics

Patients’ baseline characteristics are depicted in Table 1. Thirty-four patients (8 women; mean age: 73.1±8.5 years; body mass index 28.4±4.4) were included. For all patients, a CHA_2_DS_2_-VASC score between 1 and 6 (mean 3.6±1.2) and a HAS-BLED score between 1 and 4 (mean 2.7±0.9) were calculated. Calculated stroke and bleeding risks did not differ between the EN+ (n = 17) and EN- (n = 17) group.

**Table 1:** Baseline characteristics. ACE: angiotensin converting enzyme; AF: atrial fibrillation; INR: international normalized ratio; PCI: percutaneous coronary intervention; SD: standard deviation.

	Total (n = 34)	EN+ (n = 17)	EN- (n = 17)
**Demographics**			
Age (years), mean ± SD	73.1±8.5	73.1±9.1	72.8±8.1
Male gender, n (%)	26 (76.5)	10 (58.8)	16 (94.1)
Body mass index (kg/m^2^), mean ± SD	28.4±4.4	27.5±4.6	29.3±4.1
**Other diseases**			
Hypertension, n (%)	28 (82.4)	13 (76.5)	15 (88.2)
Congestive heart failure, n (%)	29 (85.3)	14 (82.4)	15 (88.2)
Prior stroke / transient ischemic attack, n (%)	4 (11.8)	2 (11.8)	2 (11.8)
Coronary artery disease, n (%)	21 (61.8)	9 (52.9)	12 (70.6)
Prior myocardial infarction, n (%)	11 (32.4)	4 (23.5)	7 (41.2)
Previous PCI, n (%)	16 (47.1)	7 (41.2)	9 (52.9)
Pervious cardiac surgery, n (%)	4 (11.8)	3 (17.7)	4 (23.5)
**Medical treatment prior to LAA closure**			
Acetylsalicylic acid, n (%)	33 (97.1)	16 (94.1)	17 (100)
Clopidogrel, n (%)	30 (88.2)	15 (88.2)	15 (88.2)
Phenprocoumon, n (%)	4 (11.8)	2 (11.8)	2 (11.8)
Novel oral anticoagulants, n (%)	2 (5.9)	1 (5.9)	1 (5.9)
Dual antithrombotic therapy, n (%)	30 (88.2)	15 (88.2)	15 (88.2)
Triple antithrombotic therapy, n (%)	4 (11.8)	2 (11.8)	2 (11.8)
Betablocker, n (%)	30 (88.2)	15 (88.2)	15 (88.2)
Class IV antiarrhythmics, n (%)	9 (26.5)	4 (23.5)	5 (29.4)
Digitalis glycosides, n (%)	9 (26.5)	3 (17.7)	6 (35.3)
ACE-inhibitors / sartans, n (%)	28 (82.4)	12 (70.6)	16 (47.1)
Diuretics, n (%)	18 (52.9)	9 (52.9)	99 (52.9)
Statins, n (%)	26 (76.5)	12 (70.6)	14 (82.4)
Antidiabetics, n (%)	8 (23.5)	4 (23.5)	4 (23.5)
**Echocardiography parameter**			
Left ventricular ejection fraction (%), mean ± SD	52±11	51±13	53±7
Left atrial area (cm^2^), mean ± SD	26±8	26±9	26±9
**AF pattern**			
Paroxysmal, n (%)	23 (67.7)	10 (58.8)	13 (76.5)
Persistent, n (%)	6 (17.7)	4 (23.5)	2 (11.8)
Long standing persistent, n (%)	5 (14.7)	3 (17.7)	2 (11.8)
**Scores**			
CHA_2_DS_2_ VASC 1/2/3/4/5-9, n	2/4/10/10/8	2/2/4/6/3	0/2/6/4/5
HAS BLED 0-1/2/3/4/5-9, n	3/11/13/7/0	2/5/7/3/0	1/6/6/4/0
EHRA I/II/III/IV, n	26/8/0/0	16/1/0/0	11/6/0/0
**Indication for LAA closure**			
Bleeding, n (%)	22 (64.7)	13 (76.5)	9 (52.9)
INR lability, n (%)	4 (11.8)	1 (5.9)	3 (17.7)
Non compliance, n (%)	7 (20.6)	3 (17.7)	4 (23.5)
High bleeding risk, n (%)	1 (2.9)	0 (0)	1 (5.9)

### LAA closure

All patients underwent successful LAA closure. LAA access by transseptal puncture (n = 34) was guided by TEE in 32 patients (EN+: n = 16 vs. EN-: n = 16; p = 1.0000). The “marker feature” was used in all patients in the EN+ group (for a representative image integration demonstrating the EN see S1 and S2 Moving Images). Covering of the LAA orifice by the disk of the ACP was documented by TEE and angiography in all patients in the EN+ and the EN- group after device release.

LAA measurements varied relative to the used imaging modality. The maximum LAA orifice diameter (ANOVA; F = 1.589, p = 0.2110) and length (ANOVA; F = 0.6821, p = 0.5087) did not differ between 2D-, 3D-TEE and angiography. LAA orifice area was smaller in 2D-TEE measurements (2.4±4 cm^2^) compared to 3D-TEE (2.9±1.1 cm^2^; p = 0.01) (S1 Table). The mean device size did not differ between the EN+ and the EN- group. In 5 of 34 patients (15%) devices needed to be changed during the procedure due to initial mis-sizing (2 times in the EN+ and 3 times in the EN- group).

### Primary and secondary endpoints

Total radiation dose was reduced about 52% in the EN+ group (EN+: 48.5±30.7 Gy/cm^2^ vs. EN-: 93.9±64.4 Gy/cm^2^; p = 0.01) compared to the EN- group (Table 2). Corresponding to the radiation dose fluoroscopy time was reduced (EN+: 16.7±7 minutes vs. EN-: 24.0±11.4 minutes; p = 0.035). Procedure time (EN+: 89.6±28.8 minutes vs. EN-: 90.1±30.2 minutes; p = 0.96) and contrast media amount (EN+: 172.3±92.7 ml vs. EN-: 197.5±127.8 ml; p = 0.53) did not differ between both groups (Table 2).

**Table 2:** Procedural data.

	Total (n = 34)	EN+ (n = 17)	EN- (n = 17)	p-value
Radiation dose (Gy/cm^2^), mean ± SD	70.5±54	48.5±30.7	93.9±64.4	0.01
Fluoroscopy time (min), mean ± SD	20.3±10	16.7±7	24.0±11.4	0.035
Contrast media amount (ml), mean ± SD	184.9±110.6	172.3±92.7	197.5±127.8	0.53
Procedure time (min), mean ± SD	89.9±29	89.6±28.8	90.1±30.2	0.96
Device sizes (mm), mean ± SD	25±2	25±3	26±2	0.2
Lobe diameter (mm): 22/24/26/28/30, n	6/9/11/6/1	5/3/5/3/0	1/6/6/3/1	
Major complications				
Serious pericardial effusion	-	-	-	
Systemic embolism	-	-	-	
Ischemic stroke	-	-	-	
Hemorrhagic stroke	-	-	-	
Minor complications				
Pericardial effusion, without tamponade	-	-	-	
Femoral hematoma, n (%)	2 (5.9)	1 (5.9)	1 (5.9)	
Others	-	-	-	

### Follow-Up

No periprocedural cardiovascular events and procedure or device related complications requiring major cardiovascular or endovascular intervention occurred (Table 2). During short-term follow-up of at least 3 months (mean: 8.1±5.9 months) no cerebrovascular complications occurred. Unfortunately, one multimorbid patient (female, 61 years, EN+ group) with a porcelain aorta and previous transapical aortic valve implantation died following paravalvular leak closure (Amplatzer Vascular Plug III 10 x 5 mm; AGA Medical Corporation, Plymouth, Minnesota) due to a rapidly progressive aortic regurgitation (pressure half time 440 ms, Jet/LVOT 28%). The paravalvular leak closure had to be performed due to cardiac decompensation and advanced dyspnoea. There was no evidence for dislocation or thrombus formation related to the LAA occluder. In one patient of the EN- group a slight peri-device flow was documented 3 months after LAA closure and oral anticoagulation was reinitiated due to the patient’s wish to reduce the risk of thromboembolism. During follow-up no accompanying cardiovascular events occurred.

## Discussion

The major findings of the present study are: 1. Automated real-time integration of echocardiography and fluoroscopy was found to reduce radiation dose and fluoroscopy time during percutaneous left atrial appendage closure. 2. This approach did not prolong procedure time or increase periprocedural complications.

The combination of TEE and fluoroscopy is well established for percutaneous LAA closure due to the well known limitations of each of these modalities [15,20]: 2D-TEE ensures imaging quality of relevant anatomical structures but often needs several adjustments to visualize the course of intracardiac catheters and their relationship especially with the LAA orifice and body. The latter are only partly overcome by 3D-TEE [13,16]. Fluoroscopy optimally visualizes catheters, guidewires, and devices but is limited by its 2D projections of the complex 3D anatomy of the LAA and surrounding structures. The automated real-time fusion of echocardiography and fluoroscopy combines the advantages of both imaging modalities. This might improve percutaneous LAA closure as supported by the present findings.

### Integrated echocardiography and fluoroscopy imaging during LAA closure

According to EHRA/EAPCI expert consensus on catheter-based LAA occlusion TEE is the gold standard for imaging the LAA and guiding LAA closure [21]. Nevertheless, TEE is an integral part for guidance in most but not all LAA occlusion procedures. Whether the automated real-time integration of 2D-/3D-TEE and fluoroscopic images in the same anatomical alignment might increase the number of TEE guided procedures is unknown. However, several potential benefits might be noteworthy [6]. First, landmark setting supports complex transseptal punctures [6] and LAA occluder positioning. Second, objects which are best visualized in different imaging modalities (i.e. the LAA orifice in echocardiography and the device in fluoroscopy) are directly and in real-time displayed in the same orientation. Especially during device deployment, when the anatomical target is best visualized in ultrasound and the device in fluoroscopy, the integrated imaging displays both modalities in the same perspective so that mental work is reduced for the operator. Third, integrated imaging provides in real-time several information for device deployment and stability in variable, patient-specific LAA anatomies in one image. Features like free rotation and zoom of an echocardiographic image, which are controlled by a tableside control, add additional information to TEE color doppler and angiography regarding adequate LAA sealing and device stability. Some of these advantages might also be realized by intracardiac echocardiography [22]. This cannot be answered by the present study. The wider use of the latter might be influenced by operateur preferences and its availability while the EN incorporates modalities which are available in every catheterization laboratory.

Importantly, although the integrated imaging system can facilitate the procedure for the operator, it has also a potential safety concern. A shift between both imaging modalities may occur, so that the two images will not be displayed in the same orientation any more. Although this kind of shift is visualized on the screen (see green coloring of TEE probe in Figs 2 and 3 which would turn red) procedural mistakes could occur.

### Radiation exposure

The finding that the radiation exposure was reduced by using integrated imaging suggests that the software module facilitates left atrial visualization. It is likely that integrated images of the LAA obtained with echocardiography and fluoroscopy predominantly reduced the need for multiple LAA angiograms. Whether or how a pre-procedure computer tomographic angiography or an intra-procedure C-arm rotational angiography might impact the identification of the optimal gantry position for device deployment in challenging cases was beyond the scope of the present study and needs to be determined. Although the impact of radiation dose reduction is not known, it may be relevant for several patient populations (e.g. obese and younger patients) and may in the future reduce the lifetime risk of cancer for medical staff performing fluoroscopy guided procedures over many years [23].

While radiation exposure was reduced, procedure time was similar in both groups. This might be in part explained by the fact that landmark setting and measurements during the procedure are time-consuming [6]. This is in line with findings by Sündermann and colleagues who nicely demonstrated the feasibility of using the EN during edge-to-edge mitral valve repair [6]. In addition, angiographies are relatively standardized in our approach. The resulting procedure time is still a noteworthy issue. Whether technical improvements like direct overlay might overcome this and other limitations has not been systematically investigated. It needs to be demonstrated if those upgrades might finally translate into improved outcome and patient safety.

### Procedural success and follow-up

Although early in its clinical use, we investigated the potential impact of integrated imaging on short-term outcome.

Importantly, we only included a relatively small number of patients undergoing LAA closure due to the fact that the aim of the study was to investigate whether integrated imaging reduces radiation exposure. Regarding the outcome the amount of patients is of course critical. Since the overall complication rate is expected to be higher (serious pericardial effusion about 1–2%, device embolization ∼1%, ischemic stroke ∼1%) [21] no reliable conclusion can be drawn regarding the safety of integrated imaging from the present data. Variables like anatomical variations or difficult TEE image acquisition may influence procedural success. Whether and how the presented approach might be useful to overcome technical challenges in some patients is yet not known. Further large-scale trials are warranted to ultimately assess the safety of this novel fusion imaging approach. However, there are no reasons to expect any unforeseen limitations when automated real-time image integration is used since its reliability and stability could be shown.

## Conclusion

Automated real-time integration of echocardiography and fluoroscopy can be easily incorporated into procedural work-flow of percutaneous left atrial appendage closure without prolonging procedure time. This approach results in a relevant reduction of radiation exposure for patients and medical staff.

## Supporting Information

S1 CONSORT Checklist CONSORT checklist. (DOC)

S1 Protocol Trial protocol. (DOC)

S1 Table Comparison of 2D-TEE and 3D-TEE LAA measurements. The LAA orifice area was smaller in 2D-TEE measurements (2.4±1.4 cm^2^) compared to 3D-TEE (2.9±1.1 cm^2^; p = 0.01). The maximum LAA orifice diameter (ANOVA; F = 1.589, p = 0.21) and length (ANOVA; F = 0.6821, p = 0.51) did not differ between 2D-, 3D-TEE and angiography.
(DOCX)

S1 Fig LAA characterization by using 2D- (A), 3D-TEE (B), and angiography (C). All three imaging modalities show different morphological details in a patient with nearly identical diameters of the LAA orifice. Note the LCX which is optimally visualized in 2D-TEE only. 3D-TEE derived LAA measurements have been described to be more accurate reflecting the “real” LAA anatomy and morphology. In our experience a “stepwise approach” combining different imaging modalities in a systematic manner give greater confidence during critical steps of the procedure.
(TIF)

S1 Moving Image Integrated echocardiography and fluoroscopy imaging during LAA closure. First, the conventional echocardiographic view using the x-plane mode (left panel) and the fluoroscopic view (right panel) including angiography are displayed. Outlines of 2D-TEE (right panel, pink lines) are fused with the fluoroscopy image. Next, in the four window view the 2D-TEE image (lower left panel) is in the same anatomical alignment as the C-arm (lower right panel). In a third window (upper left panel), the interventional cardiologist can simultaneously rotate and zoom an echocardiography image (“Free” view) independently from the echocardiographer by using a tableside control. In the fourth window (upper right panel), the left atrium is shown in the conventional echocardiographic views (“Echo” view). Markers demonstrating the crista and LCX have been used.
(AVI)

S2 Moving Image 3D Visualization of the left atrium during integrated imaging. Fluoroscopy (lower right) and 3D-TEE imaging are used to confirm correct device positioning. The LAA occluder is scanned in the 3D-TEE “Free view” (upper left) by using the tableside control demonstrating the relationship to surrounding structures (LCX, crista). The sheath is displayed in the upper right panel. Note the large crista, which could not be fully covered by the occluder.
(AVI)

## Abbreviations

ACP: Amplatzer^TM^ Cardiac Plug
AF: Atrial fibrillation
EHRA: European Heart Rhythm Association
EN: EchoNavigator
EN+: Procedures performed using the EchoNavigator
EN-: Procedures performed without the EchoNavigator
LAA: Left atrial appendage
LCX: Left circumflex artery
SD: Standard deviation
SJM: St. Jude Medical^®^
TEE: Transesophageal echocardiography
