# Supplementary material for: Left Atrial Appendage Closure Guided by Integrated Echocardiography and Fluoroscopy Imaging Reduces Radiation Exposure
Source: PLoS One. 2015 Oct 14;10(10):e0140386. doi: 10.1371/journal.pone.0140386 (PMC4605826; doi:10.1371/journal.pone.0140386)
Supplement: S1 Protocol — (DOC) [file pone.0140386.s002.doc]

**Klinik für Kardiologie, Pneumologie und Angiologie**

**Direktor der Klinik**

Univ.-Prof. Dr. med. M. Kelm

Clinical Study Outline

**Fusion imaging**

**during left atrial appendage closure**

Version 1.0

| **Principal Investigator** | PD Dr. Christian Meyer, MD  Klinik für Kardiologie, Pneumologie und Angiologie Moorenstr. 5 40225 Düsseldorf |
| --- | --- |
| **Sub-Investigators** | Jan Balzer, MD  Christian Eickholt, MD  Tobias Zeus, MD  Christiane Peiker, MD |

***Confidential***

# Abbreviations

| AF | Atrial fibrillation |
| --- | --- |
| LAA | Left atrial appendage |
| TEE | Trans-esophageal echocardiography |

| **Title of Study** | **Fusion imaging during left atrial appendage closure** |
| --- | --- |
| **Principal Investigator** | PD Dr. Christian Meyer |
| **Sub-Investigators** | Jan Balzer, MD  Christian Eickholt, MD  Tobias Zeus, MD  Christiane Peiker, MD |
| **Study Duration** | First patient in: Q1 2012  Recruitment Phase: 24 months Observational period per patient: 3-12 months  Last patient out: Q4 2014 |
| **Legal Aspects** | The study will be performed in accordance with the Declaration of Helsinki and the Good clinical practice (GCP)-guideline. |
| **Background** | Percutaneous left atrial appendage (LAA) closure is currently under investigation as a promising catheter-based approach for stroke prevention in patients with atrial fibrillation (AF) [1,2,3]. This is important since the LAA is the source of thrombi in >90% of affected patients with nonvalvular AF [4] while oral anticoagulation still bears several limitations including bleeding risk [2]. Importantly, LAA closure still remains technically challenging and exposes patients to a considerable risk [5]. Recently, a novel system enabling integrated echocardiography and fluoroscopy imaging (EchoNavigator®), Philips Healthcare), has been introduced [6,7]. The usefulness of this novel imaging approach during LAA closure procedures has not been investigated so far. Herein, we investigate the utility of LAA closure guided by integrated echocardiography and fluoroscopy imaging. |
| **Risk-Benefit-Assessment** | All patients with nonvalvular AF, a CHA_2_DS_2_-VASc score of ≥1 and a relative contraindication to oral anticoagulation will be assigned to LAA closure for stroke prevention. LAA closure still remains technically challenging and exposes patients to a considerable risk [5]. The EchoNavigator® system might at least partly overcome these limitations including radiation exposure [6,7]. Only patients with a medical indication for LAA closure will be included in this observational study. Standard procedures using CE certified techniques will be used, no additional risk is expected. The presented observational study is part of a study protocol aiming to characterize circulatory parameters to prevent hemodynamic instability (Clinical Trial Registration: URL: http://clinicaltrials.gov. Unique Identifier: NCT01262508). |
| **Objectives** | To investigate whether percutaneous left atrial appendage (LAA) closure guided by the EchoNavigator® system results in decreased radiation exposure.  **Primary outcome:** Primary endpoints are the change of total radiation dose and fluoroscopy time.  **Secondary outcomes:** Secondary endpoints are the change of procedure time and contrast media amount. Successful LAA closure (residual flow <5mm) and acute (7-day) occurrence of death, ischemic stroke, systemic embolism and procedure or device related complications requiring major cardiovascular or endovascular intervention are determined |
| **Study Design** | non-interventional, open-label, single-center, observational study |
| **Inclusion Criteria** | - Male and female patients aged > 18 years - Patients with nonvalvular AF, a CHA2DS2-VASc score of ≥1, a relative contraindication to oral anticoagulation, and a life expectancy of at least 2 years - Group 1: LAA closure with the guidance of automated real-time integration of 2D-/3D-trans-esophageal echocardiography (TEE) and fluoroscopy imaging (EchoNavigator®) - Group 2: LAA closure without the guidance of automated real-time integration of 2D-/3D-TEE and fluoroscopy imaging (EchoNavigator®) - Written informed consent |
| **Exclusion Criteria** | - Patients unwilling or unable to give informed consent - Underage persons |
| **Number of Patients** | All patients with nonvalvular AF, a CHA_2_DS_2_-VASc score of ≥1 and a relative contraindication to oral anticoagulation who will undergo a LAA closure procedure at the Heart Center Duesseldorf will be asked to participate in this observational study. The recruitment period will be 24 months. |
| **Intervention** | All included patients with nonvalvular AF, a CHA_2_DS_2_-VASc score of ≥1 and a relative contraindication to oral anticoagulation will undergo LAA closure with or without the Echo-navigator® System at the Heart Center Duesseldorf according to local standard. The procedure will be performed either in deep sedation using TEE and fluoroscopy for guidance. |
| **Methods** | After patients given their written informed consent the following data will be collected:   - Demographics and medical history - Physical examination and vital signs - Periprocedural in hospital data, e.g. procedure duration and radiation time - Laboratory parameters (blood samples for biochemistry and hematology analysis) - Trans-thoracic echocardiography - TEE   Follow-up visits will be performed routinely after 3, 6, and 12 months. The following outcome parameters will be collected:   - Physical examination and vital signs - Medical history |
| **Data Documentation** | All data to be collected will directly be entered into a suitable data base. |
| **Sample Size** | The prospectively calculated sample size using 2-sided *t*-test analysis aiming for a power of 95% and an alpha of 0.05 was 14 patients per group. With a drop-out rate of 15% we assumed a group size of 17 patients. |
| **Analysis and Statistics** | Continuous data will be expressed as mean ± standard deviation and compared with the unpaired student`s t-test or Mann-Whitney U test if not normally distributed. Normality will be checked with the Kolmogorov-Smirnov test. To compare multiple subgroups of normally distributed data a one-way ANOVA will be used. Statistical analysis will be performed with Prism® (GraphPad®). A p-value <0.05 will be considered to be significant. |
| **Ethics and Quality Assurance** | The study was approved by the local ethics committee. The study will be conducted according to the ICH-GCP criteria of ”Good Clinical Practice“. It will be performed according to the legal data protection requirements. The pseudonymized data will be archived for 5 years at the University Hospital Duesseldorf. Data analysis will be performed pseudonymized. Data protocol and publication will be anonymized. The patients have the right to receive information. |
| **Insurance** | No patients’ insurance is necessary because this is an observational trial. |

**Literature**

[1] Reddy VY, Möbius-Winkler S, Miller MA, Neuzil P, Schuler G, Wiebe J, et al. Left atrial appendage closure with the watchman device in patients with a contraindication for oral anticoagulation. J Am Coll Cardiol. 2013;61: 2551–2556.

[2] Landmesser U, Holmes DR. Left atrial appendage closure: a percutaneous transcatheter approach for stroke prevention in atrial fibrillation. Eur Heart J. 2012;33: 698–704.

[3] Lockwood SM, Alison JF, Obeyesekere MN, Mottram PM. Imaging the left atrial appendage prior to, during, and after occlusion. J Am Coll Cardiol Img. 2011;4: 303–306.

[4] Stoddard MF, Dawkins PR, Prince CR, Ammash NM. Left atrial appendage thrombus is not uncommon in patients with acute atrial fibrillation and a recent embolic event: a transesophageal echocardiographic study. J Am Coll Cardiol. 1995;25: 452–459.

[5] Reddy VY, Sievert H, Halperin J, Doshi SK, Buchbinder M, Neuzil P, et al. Percutaneous left atrial appendage closure vs warfarin for atrial fibrillation. JAMA. 2014;312: 1988-1998.

[6] Sündermann SH, Biaggi P, Grünenfelder J, Gessat M, Felix C, Bettex D, Falk V, et al. Safety and feasibility of novel technology fusing echocardiography and fluoroscopy images during MitraClip interventions. EuroIntervention. 2014;9: 1210–1216.

[7] Gafoor S, Schulz P, Heuer L, Matic P, Franke J, Bertog S, et al. Use of EchoNavigator, a novel echocardiography-fluoroscopy overlay system, for transseptal puncture and left atrial appendage occlusion. J Interv Cardiol. 2015;28: 215-217.

**Study Flow Chart**

Table 1: Visit Schedule

| **Procedure** | **Screening/**  **Baseline Visit**  **V1** | **Procedure**  **V2** | **Follow-up Visits**  **V3** |
| --- | --- | --- | --- |
| Informed Consent | X |  |  |
| In-/Exclusion Criteria | X |  |  |
| Physical Examination | X |  | X |
| Medical History | X |  | X |
| Vital Signs | X |  | X |
| TTE | X |  |  |
| TEE | X | X |  |
| Periprocedural data (e.g. procedure time, radiation time) |  | X |  |
| Laboratory analysis* | X |  |  |
| Adverse event assessment |  | X | X |

* GFR, Creatinine, Urea, Protein, Albumin, Creatine kinase, Lactate dehydrogenase, Troponin, INR, Prothrombin time, Partial thromboplastin time, Fibrinogen, BNP, Blood count, Blood glucose.

This clinical investigation plan was subject to critical review and has been approved by the following:

**Principal Investigator**

I agree that this Clinical Study Protocol contains all the information required to conduct this study. By my signature below, I hereby certify that I have read, understood and agree to abide by all conditions, instructions and restrictions contained in this clinical investigation plan.

**Principal Investigator**

Date name
