# Supplementary material for: Left Atrial Appendage Closure Guided by Integrated Echocardiography and Fluoroscopy Imaging Reduces Radiation Exposure
Source: PLoS One. 2015 Oct 14;10(10):e0140386. doi: 10.1371/journal.pone.0140386 (PMC4605826; doi:10.1371/journal.pone.0140386)
Supplement: S1 Table — The LAA orifice area was smaller in 2D-TEE measurements (2.4±1.4 cm2) compared to 3D-TEE (2.9±1.1 cm2; p = 0.01). The maximum LAA orifice diameter (ANOVA; F = 1.589, p = 0.21) and length (ANOVA; F = 0.6821, p = 0.51) did not differ between 2D-, 3D-TEE and angiography. (DOCX) [file pone.0140386.s003.docx]

|  | 2D-TEE | 3D-TEE | Angio- graphy | p-value |
| --- | --- | --- | --- | --- |
| LAA orifice area (cm²), mean ± SD | 2.4±1.4 | 2.9±1.1 | - | 0.01 |
| Maximum LAA orifice diameter (cm), mean ± SD | 2.0±0.6 | 2.3±0.5 | 2.2±0.2 | 0.21 |
| Depth (cm), mean ± SD | 3.0±8 | 2.9±7 | 3.1±0.4 | 0.51 |
