# Supplementary figures and images for: Left Atrial Appendage Closure Guided by Integrated Echocardiography and Fluoroscopy Imaging Reduces Radiation Exposure
Source: PLoS One. 2015 Oct 14;10(10):e0140386. doi: 10.1371/journal.pone.0140386 (PMC4605826; doi:10.1371/journal.pone.0140386)

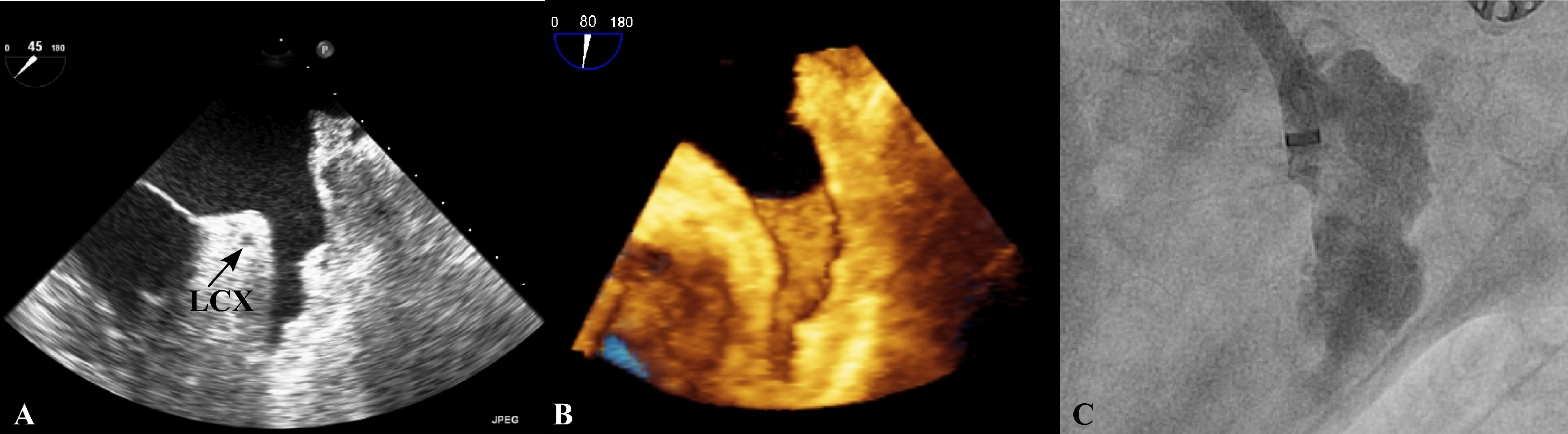

Supplement: S1 Fig — All three imaging modalities show different morphological details in a patient with nearly identical diameters of the LAA orifice. Note the LCX which is optimally visualized in 2D-TEE only. 3D-TEE derived LAA measurements have been described to be more accurate reflecting the “real” LAA anatomy and morphology. In our experience a “stepwise approach” combining different imaging modalities in a systematic manner give greater confidence during critical steps of the procedure. (TIF) [file pone.0140386.s004.tif]
